# Uric Acid and Impairment of Renal Function in Non-diabetic Hypertensive Patients

**DOI:** 10.3389/fmed.2021.746886

**Published:** 2022-01-24

**Authors:** Yi-Hsin Hung, Chin-Chou Huang, Liang-Yu Lin, Jaw-Wen Chen

**Affiliations:** ^1^Department of Internal Medicine, National Taiwan University Hospital, Taipei, Taiwan; ^2^Division of Cardiology, Department of Medicine, Taipei Veterans General Hospital, Taipei, Taiwan; ^3^School of Medicine, National Yang Ming Chiao Tung University, Taipei, Taiwan; ^4^Institute of Pharmacology, School of Medicine, National Yang Ming Chiao Tung University, Taipei, Taiwan; ^5^Cardiovascular Research Center, National Yang Ming Chiao Tung University, Taipei, Taiwan; ^6^Division of Endocrinology and Metabolism, Department of Medicine, Taipei Veterans General Hospital, Taipei, Taiwan; ^7^Healthcare and Service Center, Taipei Veterans General Hospital, Taipei, Taiwan

**Keywords:** Chinese, hypertension, renal function, nephropathy, uric acid

## Abstract

Hyperuricemia is a risk factor for renal impairment. However, investigations focusing on patients with hypertension are limited and inconsistent. A single-center prospective cohort study of 411 Han Chinese non-diabetic hypertensive patients was conducted in Taiwan. The mean age of the participants was 62.0 ±14.4 years. The baseline estimated glomerular filtration rate and uric acid level were 86 mL/min/1.73 m^2^ and 6.2 mg/dL, respectively. All patients underwent serum biochemistry tests for creatinine levels every 3 months. Renal events were defined as >25% and >50% decline in estimated glomerular filtration rate. During an average follow-up period of 4.7 ± 2.9 years (median 4.0 years), a >25 and >50% decline in estimated glomerular filtration rate was noted in 52 and 11 patients, respectively. The multivariate Cox regression analysis revealed that a baseline uric acid level ≥8.0 mg/dL increased the risk of >25% decline (hazard ratio: 3.541; 95% confidence interval: 1.655–7.574, *P* = 0.001) and >50% decline (hazard ratio: 6.995; 95% confidence interval: 1.309–37.385, *P* = 0.023) in estimated glomerular filtration rate. Similarly, a baseline uric acid level ≥7.5 mg/dL was independently associated with >25% decline (hazard ratio: 2.789; 95% confidence interval: 1.399–5.560, *P* = 0.004) and >50% decline (hazard ratio: 6.653; 95% confidence interval: 1.395–31.737, *P* = 0.017). However, this was not demonstrated at baseline uric acid level ≥7.0 mg/dL. Our study suggests that hyperuricemia is an independent risk factor for the decline in renal function in patients with hypertension. Uric acid level ≥7.5 mg/dL may be considered as the optimal cutoff value for clinical practice in predicting the development of renal impairment.

## Introduction

Hypertension is a leading cause of chronic kidney disease (CKD) (1, 2). In addition to achieving blood pressure (BP) control, it is important to identify other possible risk factors to delay the development and progression of CKD.

Previous epidemiological studies on the general population have indicated an independent effect of hyperuricemia on the risk of developing CKD (3, 4). Several studies have focused on different subpopulations, such as patients with diabetic nephropathy and IgA nephropathy (5–7). However, evidence regarding the relationship between uric acid (UA) and renal outcomes in hypertensive patients is limited and inconsistent (8, 9).

The physicochemical definition of hyperuricemia is based on the solubility limit of UA in serum (10). On the other hand, the statistical definition proposed by the American College of Rheumatology is UA above the mean plus two standard deviations for the healthy population (11). Based on the above definition, there is no universally accepted threshold and several cutoff values have been suggested, for example, >7.7 mg/dL in men and >6.6 mg/dL in women, or >7.0 mg/dL in men and >6.0 mg/dL in women (10, 12).

The guidelines of the American College of Rheumatology and the European Alliance of Associations for Rheumatology both propose the goal of managing patients with gout. However, they do not directly address the impact of hyperuricemia on renal diseases and hypertensive patients (13, 14). In addition, there is no clear UA cutoff associated with the risk of renal impairment. Whether screening of UA levels in hypertensive patients provides information for predicting and preventing renal diseases requires further research.

The present study focused on non-diabetic hypertensive patients and investigated the relationship between baseline serum UA levels and decline in renal function. In addition, we aimed to assess the serum UA cutoff value for predicting CKD development.

## Materials and Methods

### Participants

Han Chinese patients with hypertension were included in our study from February 2012 to January 2021. The inclusion criteria were as follows: patients aged ≥20 years; those of Han Chinese descent; those who are official residents in Taiwan; those meeting one of the following hypertension criteria: (a) systolic blood pressure (SBP) ≥140 mmHg or diastolic blood pressure (DBP) ≥90 mmHg in at least two consecutive visits within 2 months and (b) taking one or more antihypertensive medications; those with no medical history of severe diseases, including liver, renal, cardiac, and pulmonary failure and carcinoma; and those without acute disease within 2 weeks.

The exclusion criteria were as follows: the subject was identified as a secondary hypertension patient, unable to understand or give informed consent, and had one or more foreign parents. Patients with severe renal disease, defined as CKD stage 5 and end stage renal disease (ESRD), were excluded. Patients with diabetes mellitus and those who received uric acid-lowering agents within 3 months prior to the enrollment or during the study period were also excluded in the present study.

The study protocol was approved by the Ethics Committee of Taipei Veterans General Hospital. This study was conducted in accordance with the principles of the Declaration of Helsinki.

### Study Design

The study included a comprehensive evaluation of each participant's medical history and physical examination at the hypertension clinic of the hospital. The patients' office BP was measured, and their body mass indices (BMI) were determined. Antihypertensive drug prescriptions were recorded once they were present. All patients were followed up every 3 months.

### Office BP Measurement

According to a standardized protocol, a well-trained nurse assessed the morning office BP using an electronic BP monitor (Omron HEM-7121, Omron Healthcare Taiwan Co., Songshan, Taipei, Taiwan, ROC) after the patients were instructed to sit for 10 min in a quiet room. During each measurement, both SBP and DBP were recorded. Three consecutive BP measurements were performed in the same upper arm. Each measurement was separated at an interval of 30 s. The average value of the last two measurements was considered the BP reading.

### Laboratory Measurements

Fasting whole blood samples of the patients were obtained by venipuncture after a 10 min rest in a supine position in the morning, typically between 0730 and 0900 h. The participants were instructed to take all routine medications, as they normally would. The blood samples were centrifuged, and the serum was thawed for analysis. Serum levels of total cholesterol, triglyceride, high-density lipoprotein cholesterol (HDLC), low-density lipoprotein cholesterol (LDLC), fasting blood glucose, creatinine, and UA were measured. Patients were further divided into different groups according to baseline UA levels (≥8.0, 7.5, or 7.0 mg/dL). Kidney function was assessed by serum creatinine at baseline and every 3 months thereafter. The estimated glomerular filtration rate (eGFR) was calculated using the four-variable equation proposed by the Modification of Diet in Renal Disease Study (15).

### Renal Outcomes

Renal events during the follow-up period were defined as >25% decline or >50% decline in eGFR, which has been used to indicate minor or major renal dysfunction in previous studies (16, 17).

### Statistical Analysis

Statistical analysis was performed using the Statistical Package for Social Sciences software (version 21.0, SPSS Inc., Chicago, IL, USA). All data are expressed as the mean ± standard deviation or frequency (percentage). Survival analysis was assessed using the Kaplan–Meier curve, with significance based on the log-rank test. To assess the independent effects of UA (baseline UA ≥8.0, 7.5, or 7.0 mg/dL) and renal outcomes, Cox proportional hazard regression analysis was performed. The adjusted hazard ratios (HRs) with 95% confidence intervals (CIs) were estimated after adjusting for potential confounding factors, including age, sex, BMI, office SBP, use of antihypertensive drugs, use of furosemide, HDLC, and baseline eGFR. Statistical significance was defined as a two-sided *P* < 0.05.

## Results

The study enrolled 411 non-diabetic hypertensive participants in Taiwan. The mean age of the participants was 62.0 ± 14.4 years, and 53.8% were men. There were 4.4% of the participants being smokers. The mean BMI was 26.1 ± 3.9 kg/m^2^. The mean office SBP and DBP were 131.4 ± 16.9 and 81.6 ± 10.4 mmHg, respectively. The baseline UA level was 6.2 ± 1.5 mg/dL. The renal function of the participants upon enrollment was serum creatinine level of 0.9 ± 0.2 mg/dL and eGFR of 86.0 ± 19.4 mL/min/1.73 m^2^. The lipid profiles were as the followings, mean total cholesterol being 188.1 ± 31.4 mg/dL, triglyceride being 128.8 ± 92.6 mg/dL, HDLC being 49.4 ± 13.0 mg/dL, and LDLC being 115.5 ± 27.4 mg/dL. The use of antihypertensive medications included angiotensin-converting enzyme inhibitors (ACEIs)/angiotensin receptor blockers (ARBs) (64.2%), β-blockers (22.4%), calcium channel blockers (73.2%), and thiazide (21.2%). The use of other diuretics included spironolactone (1.5%) and furosemide (1.7%). There were 10.0, 17.6, and 2.4% of the participants taking aspirin, statins and fibrate, respectively (Table 1).

**Table 1:** Baseline characteristics.

	**All (*n =* 411)**		**All (*n =* 411)**
Age, years	62.0 ± 14.4	Aspirin, *n* (%)	41 (10.0%)
Male, *n* (%)	221 (53.8%)	Statins, *n* (%)	72 (17.6%)
BMI, kg/m^2^	26.1 ± 3.9	Fibrate, *n* (%)	10 (2.4%)
Office SBP, mmHg	131.4 ± 16.9	Total cholesterol, mg/dL	188.1 ± 31.4
Office DBP, mmHg	81.6 ± 10.4	Triglyceride, mg/dL	128.8 ± 92.6
Office HR, bpm	70.8 ± 11.1	HDLC, mg/dL	49.4 ± 13.0
Smoking, *n* (%)	18 (4.4%)	LDLC, mg/dL	115.5 ± 27.4
ACEI/ARB, *n* (%)	264 (64.2%)	Fasting blood glucose, mg/dL	98.7 ± 12.5
β-blocker, *n* (%)	92 (22.4%)	UA, mg/dL	6.2 ± 1.5
CCB, *n* (%)	301 (73.2%)	Creatinine, mg/dL	0.9 ± 0.2
Thiazide, *n* (%)	87 (21.2%)	eGFR, mL/min /1.73 m^2^	86.0 ± 19.4
Spironolactone, *n* (%)	6 (1.5%)	Follow-up duration, years	4.7 ± 2.9
Furosemide, *n* (%)	7 (1.7%)		

*ACEI, angiotensin converting enzyme inhibitor; ARB, angiotensin receptor blocker; BMI, body mass index; CCB, calcium channel blocker; DBP, diastolic blood pressure; eGFR, estimated glomerular filtration rate; HDLC, high density lipoprotein cholesterol; HR, heart rate; LDLC, low density lipoprotein cholesterol; SBP, systolic blood pressure; UA, uric acid*.

When compared to those with lower baseline UA levels, patients with higher baseline UA levels were more likely to be male, have a higher BMI, have a worse renal function, have a lower HDLC, and use thiazide and furosemide (Tables 2–4).

**Table 2:** Baseline characteristics according to uric acid levels (≥8.0 mg/dL).

	**UA <8.0 mg/dL (*n =* 365)**	**UA ≥8.0 mg/dL (*n =* 46)**	***P*-value**
Age, years	61.9 ± 13.9	62.5 ± 18.0	0.787
Male, *n* (%)	188 (51.5%)	33 (71.7%)	0.009
BMI, kg/m^2^	26.0 ± 3.8	26.8 ± 4.6	0.264
Office SBP, mmHg	131.1 ± 16.6	133.8 ± 19.3	0.370
Office DBP, mmHg	81.8 ± 10.4	80.5 ± 10.4	0.428
Office HR, bpm	71.1 ± 11.1	68.3 ± 10.6	0.093
Smoking, *n* (%)	14 (3.8%)	4 (8.7%)	0.130
ACEI/ARB, *n* (%)	229 (62.7%)	35 (76.1%)	0.075
β-blocker, *n* (%)	82 (22.5%)	10 (21.7%)	0.911
CCB, *n* (%)	267 (73.2%)	34 (73.9%)	0.912
Thiazide, *n* (%)	70 (19.2%)	17 (37.0%)	0.005
Spironolactone, *n* (%)	5 (1.4%)	1 (2.2%)	0.512
Furosemide, *n* (%)	4 (1.1%)	3 (6.5%)	0.033
Aspirin, *n* (%)	35 (9.6%)	6 (13.0%)	0.437
Statins, *n* (%)	67 (18.4%)	5 (10.9%)	0.206
Fibrate, *n* (%)	9 (2.5%)	1 (2.2%)	>0.999
Total cholesterol, mg/dL	188.4 ± 31.8	186.4 ± 28.7	0.667
Triglyceride, mg/dL	125.3 ± 89.3	156.6 ± 112.7	0.075
HDLC, mg/dL	50.0 ± 13.4	44.7 ± 7.2	0.008
LDLC, mg/dL	115.7 ± 27.6	114.0 ± 25.7	0.681
Fasting blood glucose, mg/dL	98.7 ± 12.7	98.5 ± 10.8	0.934
UA, mg/dL	5.8 ± 1.1	9.1 ± 0.8	<0.001
Creatinine, mg/dL	0.8 ± 0.2	1.0 ± 0.3	<0.001
eGFR, mL/min /1.73 m^2^	87.3 ± 18.8	76.0 ± 20.7	0.001
Follow-up duration, years	4.7 ± 2.9	4.9 ± 2.8	0.557

*ACEI, angiotensin converting enzyme inhibitor; ARB, angiotensin receptor blocker; BMI, body mass index; CCB, calcium channel blocker; DBP, diastolic blood pressure; eGFR, estimated glomerular filtration rate; HDLC, high density lipoprotein cholesterol; HR, heart rate; LDLC, low density lipoprotein cholesterol; SBP, systolic blood pressure; UA, uric acid*.

**Table 3:** Baseline characteristics according to uric acid level (≥7.5 mg/dL).

	**UA <7.5 mg/dL (*n =* 335)**	**UA ≥7.5 mg/dL (*n =* 76)**	***P*-value**
Age, years	62.3 ± 13.8	60.5 ± 17.0	0.317
Male, *n* (%)	163 (48.7%)	58 (76.3%)	<0.001
BMI, kg/m^2^	25.9 ± 3.8	27.2 ± 4.4	0.022
Office SBP, mmHg	131.1 ± 16.4	132.7 ± 18.9	0.481
Office DBP, mmHg	81.8 ± 10.2	80.8 ± 11.3	0.465
Office HR, bpm	70.8 ± 10.8	70.6 ± 12.2	0.885
Smoking, *n* (%)	13 (3.9%)	5 (6.6%)	0.347
ACEI/ARB, *n* (%)	208 (62.1%)	56 (73.7%)	0.057
β-blocker, *n* (%)	76 (22.7%)	16 (21.1%)	0.758
CCB, *n* (%)	245 (73.1%)	56 (73.7%)	0.922
Thiazide, *n* (%)	60 (17.9%)	27 (35.5%)	0.001
Spironolactone, *n* (%)	4 (1.2%)	2 (2.6%)	0.307
Furosemide, *n* (%)	4 (1.2%)	3 (3.9%)	0.121
Aspirin, *n* (%)	33 (9.9%)	8 (10.5%)	0.859
Statins, *n* (%)	64 (19.2%)	8 (10.5%)	0.074
Fibrate, *n* (%)	8 (2.4%)	2 (2.6%)	>0.999
Total cholesterol, mg/dL	188.2 ± 31.8	187.7 ± 29.6	0.881
Triglyceride, mg/dL	125.0 ± 91.8	145.7 ± 94.8	0.088
HDLC, mg/dL	50.5 ± 13.5	45.0 ± 8.8	0.001
LDLC, mg/dL	115.1 ± 27.6	117.1 ± 26.5	0.556
Fasting blood glucose, mg/dL	98.5 ± 12.5	99.3 ± 12.5	0.649
UA, mg/dL	5.7 ± 1.0	8.5 ± 1.0	<0.001
Creatinine, mg/dL	0.8 ± 0.2	1.0 ± 0.2	<0.001
eGFR, mL/min /1.73 m^2^	87.7 ± 19.2	78.6 ± 18.4	<0.001
Follow-up duration, years	4.7 ± 2.9	4.7 ± 2.7	0.918

*ACEI, angiotensin converting enzyme inhibitor; ARB, angiotensin receptor blocker; BMI, body mass index; CCB, calcium channel blocker; DBP, diastolic blood pressure; eGFR, estimated glomerular filtration rate; HDLC, high density lipoprotein cholesterol; HR, heart rate; LDLC, low density lipoprotein cholesterol; SBP, systolic blood pressure; UA, uric acid*.

**Table 4:** Baseline characteristics according to uric acid level (≥7.0 mg/dL).

	**UA <7.0 mg/dL (*n =* 296)**	**UA ≥7.0 mg/dL (*n =* 115)**	***P*-value**
Age, years	62.3 ±13.6	61.1 ± 16.3	0.441
Male, *n* (%)	131 (44.3%)	90 (78.3%)	<0.001
BMI, kg/m^2^	25.8 ± 3.7	27.0 ± 4.3	0.008
Office SBP, mmHg	130.6 ± 16.2	133.3 ± 18.4	0.173
Office DBP, mmHg	81.6 ± 10.2	81.6 ± 10.8	0.948
Office HR, bpm	70.6 ± 10.6	71.2 ± 12.3	0.693
Smoking, *n* (%)	12 (4.1%)	6 (5.2%)	0.605
ACEI/ARB, *n* (%)	178 (60.1%)	86 (74.8%)	0.005
β-blocker, *n* (%)	64 (21.6%)	28 (24.3%)	0.552
CCB, *n* (%)	216 (73.0%)	85 (73.9%)	0.847
Thiazide, *n* (%)	45 (15.2%)	42 (36.5%)	<0.001
Spironolactone, *n* (%)	4 (1.4%)	2 (1.7%)	0.674
Furosemide, *n* (%)	4 (1.4%)	3 (2.6%)	0.405
Aspirin, *n* (%)	26 (8.8%)	15 (13.0%)	0.196
Statins, *n* (%)	59 (20.0%)	13 (11.3%)	0.038
Fibrate, *n* (%)	8 (2.7%)	2 (1.7%)	0.732
Total cholesterol, mg/dL	186.7 ± 32.1	191.8 ± 29.3	0.125
Triglyceride, mg/dL	119.1 ± 80.4	153.7 ± 115.1	0.001
HDLC, mg/dL	51.0 ± 13.7	45.5 ± 9.9	<0.001
LDLC, mg/dL	114.2 ± 28.0	118.8 ± 25.5	0.110
Fasting blood glucose, mg/dL	97.8 ± 11.9	100.9 ± 13.7	0.023
UA, mg/dL	5.5 ± 0.9	8.1 ± 1.0	<0.001
Creatinine, mg/dL	0.8 ± 0.2	1.0 ± 0.2	<0.001
eGFR, mL/min /1.73 m^2^	88.4 ± 19.5	79.8 ± 17.6	<0.001
Follow-up duration, years	4.7 ± 2.9	4.7 ± 2.8	0.836

*ACEI, angiotensin converting enzyme inhibitor; ARB, angiotensin receptor blocker; BMI, body mass index; CCB, calcium channel blocker; DBP, diastolic blood pressure; eGFR, estimated glomerular filtration rate; HDLC, high density lipoprotein cholesterol; HR, heart rate; LDLC, low density lipoprotein cholesterol; SBP, systolic blood pressure; UA, uric acid*.

During a mean follow-up period of 4.7 ± 2.9 years (median 4.0 years), a >25% and >50% decline in eGFR was noted in 52 and 11 patients, respectively. Participants with higher baseline UA levels had higher rates of renal events than their counterparts. A statistically significant increase in the incidence of >25% decline in eGFR was observed if the baseline UA was ≥8.0 mg/dL (*P* = 0.004) or ≥7.5 mg/dL (*P* = 0.040). Moreover, a statistically significant increase in the incidence of >50% decline in eGFR was observed if the baseline UA was ≥8.0 mg/dL (*P* = 0.025) or ≥7.5 mg/dL (*P* = 0.035) (Table 5).

**Table 5:** Uric acid and renal events.

	**Patient number**	**>25% decline in eGFR, *n* (%)**	***P*-value**	**>50% decline in eGFR, *n* (%)**	***P*-value**
**UA levels**					
<8.0 mg/dL	365	40 (11.0%)	0.004	7 (1.9%)	0.025
≥8.0 mg/dL	46	12 (26.1%)		4 (8.7%)	
**UA levels**					
<7.5 mg/dL	335	37 (11.0%)	0.040	6 (1.8%)	0.035
≥7.5 mg/dL	76	15 (19.7%)		5 (6.6%)	
**UA levels**					
<7.0 mg/dL	296	35 (11.8%)	0.418	6 (2.0%)	0.191
≥7.0 mg/dL	115	17 (14.8%)		5 (4.3%)	

*eGFR, estimated glomerular filtration rate; UA, uric acid*.

**Figure 1:**
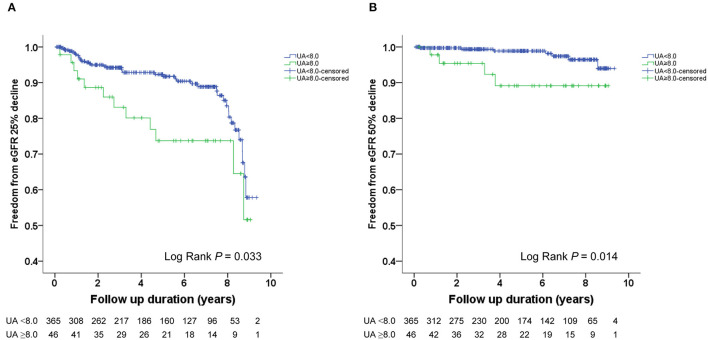
Kaplan-Meier survival curves showing the absence of renal events according to the baseline uric acid (UA) in patients with hypertension. All participants were divided into two groups according to UA levels. The blue line represents the patient group with UA <8.0 mg/dL. The green line represents the group with UA ≥8.0 mg/dL. Renal events were defined as >25% decline and >50% decline in eGFR. Differences were compared using the log-rank test. **(A)** UA (<8.0 vs. ≥8.0 mg/dL) and >25% decline in eGFR (*P* = 0.033). **(B)** UA (<8.0 vs. ≥8.0 mg/dL) and >50% decline in eGFR (*P* = 0.014).

**Figure 2:**
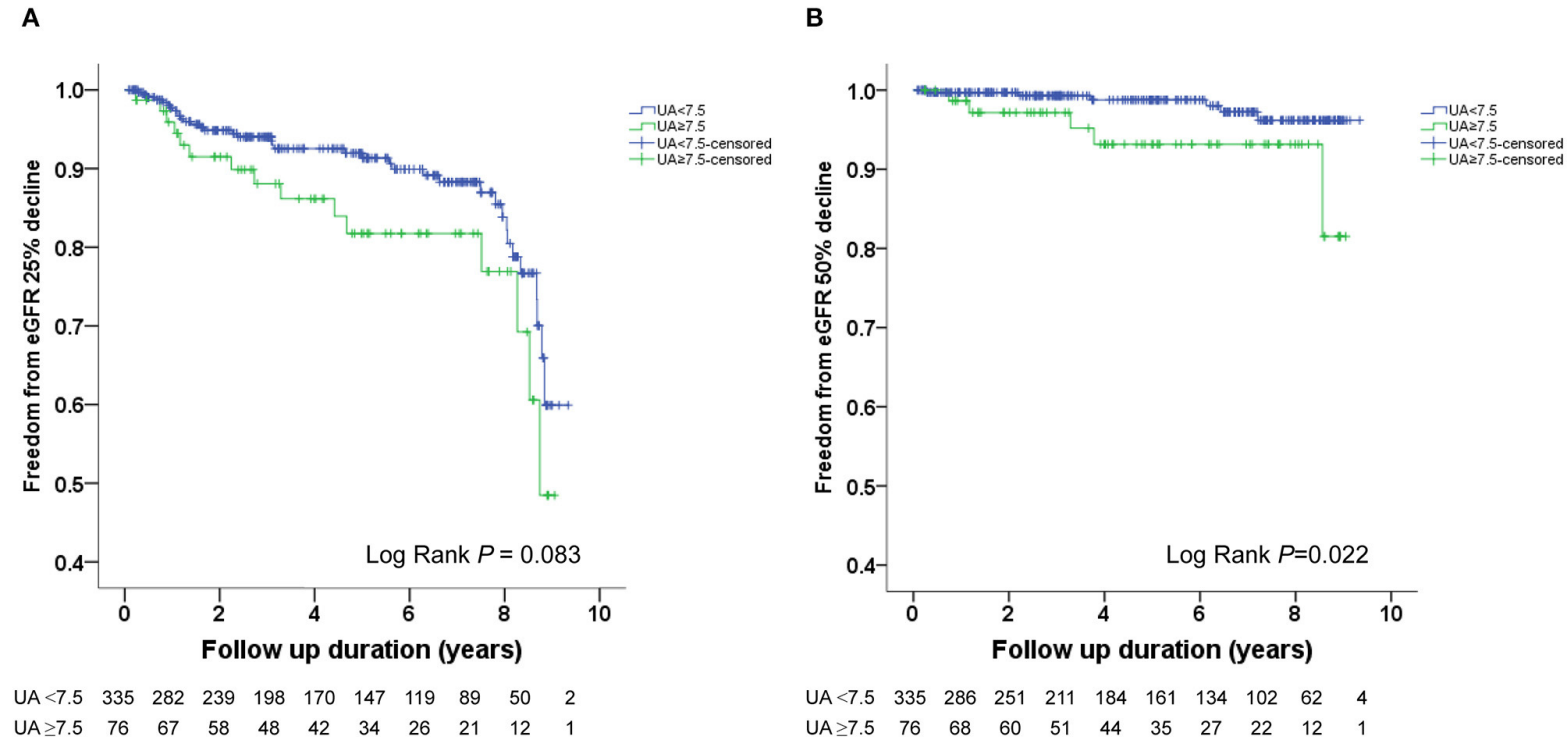
Kaplan-Meier survival curves showing the absence of renal events according to the baseline uric acid (UA) in patients with hypertension. All participants were divided into two groups according to UA levels. The blue line represents the patient group with UA <7.5 mg/dL. The green line represents the group with UA ≥7.5 mg/dL. Renal events were defined as >25% decline and >50% decline in eGFR. Differences were compared using the log-rank test. **(A)** UA (<7.5 vs. ≥7.5 mg/dL) and >25% decline in eGFR (*P* = 0.083). **(B)** UA (<7.5 vs. ≥7.5 mg/dL) and >50% decline in eGFR (*P* = 0.022).

**Figure 3:**
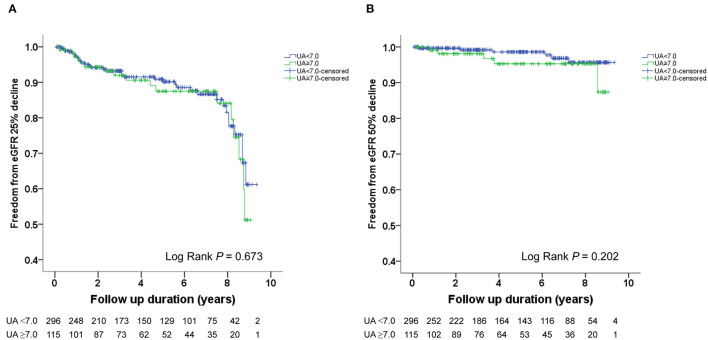
Kaplan-Meier survival curves showing the absence of renal events according to the baseline uric acid (UA) in patients with hypertension. All participants were divided into two groups according to UA levels. The blue line represents the patient group with UA <7.0 mg/dL. The green line represents the group with UA ≥7.0 mg/dL. Renal events were defined as >25% decline and >50% decline in eGFR. Differences were compared using the log-rank test. **(A)** UA (<7.0 vs. ≥7.0 mg/dL) and >25% decline in eGFR (*P* = 0.673). **(B)** UA (<7.0 vs. ≥7.0 mg/dL) and >50% decline in eGFR (*P* = 0.202).

The Kaplan–Meier survival curves and log-rank test were used to identify the number of participants who did not develop renal impairment during the follow-up period. The incidence of renal events (>25% and >50% decline in eGFR) was significantly higher in patients with a baseline UA level ≥8.0 mg/dL (*P* = 0.033 and 0.014, respectively) (Figures 1A,B). Similarly, the participants who presented with baseline UA ≥7.5 mg/dL had more renal events (>50% decline in eGFR) (*P* = 0.022) (Figures 2A,B). However, the participants who presented with baseline UA ≥7.0 mg/dL during the initial visit had similar renal events (>25% and >50% decline in eGFR) (*P* = 0.673 and 0.202, respectively) (Figures 3A,B).

Multivariate Cox regression analysis revealed that a baseline UA level ≥8.0 mg/dL was independently associated with a risk of >25% decline in eGFR (HR: 3.541; 95% CI: 1.655–7.574, *P* = 0.001) and a >50% decline in eGFR (HR: 6.995; 95% CI: 1.309–37.385, *P* = 0.023) (Table 6). Similarly, a baseline UA level ≥7.5 mg/dL was independently associated with a >25% decline in eGFR (HR: 2.789; 95% CI: 1.399–5.560, *P* = 0.004) and >50% decline in eGFR (HR: 6.653; 95% CI: 1.395–31.737, *P* = 0.017) (Table 7). However, a baseline UA level ≥7.0 mg/dL was not associated with a >25% decline in eGFR (HR: 1.577; 95% CI: 0.803–3.095, *P* = 0.186) or >50% decline in eGFR (HR: 2.756; 95% CI: 0.607–12.519, *P* = 0.189) (Table 8).

**Table 6:** Uric acid 8.0 mg/dL and decline of estimated glomerular filtration rate (eGFR).

**eGFR >25% reduction**	**HR**	**(95% CI)**	***P*-value**	**eGFR >50% reduction**	**HR**	**(95% CI)**	***P*-value**
**Univariate analysis**				**Univariate analysis**			
UA ≥8.0 mg/dL (yes vs. no)	1.997	(1.043–3.820)	0.037	UA ≥8.0 mg/dL (yes vs. no)	4.151	(1.213–14.201)	0.023
**Multivariate analysis**				**Multivariate analysis**			
Age, years	1.036	(1.009–1.063)	0.007	Age, years	1.023	(0.959–1.091)	0.489
Sex (male vs. female)	0.738	(0.387–1.409)	0.357	Sex (male vs. female)	0.273	(0.063–1.186)	0.083
BMI, kg/m^2^	0.998	(0.917–1.086)	0.957	BMI, kg/m^2^	0.923	(0.755–1.128)	0.433
Office SBP, mmHg	1.021	(1.004–1.038)	0.017	Office SBP, mmHg	1.056	(1.014–1.099)	0.008
ACEI/ARB (yes vs. no)	0.572	(0.303–1.079)	0.085	ACEI/ARB (yes vs. no)	0.474	(0.106–2.122)	0.329
β-blocker (yes vs. no)	1.605	(0.873–2.952)	0.128	β-blocker (yes vs. no)	1.961	(0.474–8.125)	0.353
CCB (yes vs. no)	1.279	(0.638–2.562)	0.488	CCB (yes vs. no)	0.412	(0.096–1.765)	0.232
Thiazide (yes vs. no)	1.156	(0.597–2.238)	0.667	Thiazide (yes vs. no)	2.116	(0.541–8.274)	0.281
Furosemide (yes vs. no)	0.782	(0.097–6.289)	0.817	Furosemide (yes vs. no)	14.990	(1.021–220.070)	0.048
HDLC, mg/dL	0.988	(0.962–1.014)	0.365	HDLC, mg/dL	0.963	(0.904–1.026)	0.243
eGFR, mL/min /1.73 m^2^	1.024	(1.007–1.042)	0.005	eGFR, mL/min /1.73 m^2^	1.026	(0.990–1.064)	0.157
UA ≥8.0 mg/dL (yes vs. no)	3.541	(1.655–7.574)	0.001	UA ≥8.0 mg/dL (yes vs. no)	6.995	(1.309–37.385)	0.023

*ACEI, angiotensin converting enzyme inhibitor; ARB, angiotensin receptor blocker; BMI, body mass index; CCB, calcium channel blocker; CI, confidence interval; eGFR, estimated glomerular filtration rate; HDLC, high density lipoprotein cholesterol; HR, hazard ratio; SBP, systolic blood pressure; UA, uric acid*.

**Table 7:** Uric acid 7.5 mg/dL and decline of estimated glomerular filtration rate (eGFR).

**eGFR >25% reduction**	**HR**	**(95% CI)**	***P*-value**	**eGFR >50% reduction**	**HR**	**(95% CI)**	***P*-value**
**Univariate analysis**				**Univariate analysis**			
UA ≥7.5 mg/dL (yes vs. no)	1.690	(0.927–3.081)	0.087	UA ≥7.5 mg/dL (yes vs. no)	3.658	(1.115–12.002)	0.032
**Multivariate analysis**				**Multivariate analysis**			
Age, years	1.036	(1.009–1.064)	0.008	Age, years	1.024	(0.960–1.092)	0.473
Sex (male vs. female)	0.724	(0.383–1.369)	0.320	Sex (male vs. female)	0.273	(0.066–1.136)	0.074
BMI, kg/m^2^	0.993	(0.911–1.083)	0.879	BMI, kg/m^2^	0.897	(0.731–1.100)	0.297
Office SBP, mmHg	1.021	(1.004–1.038)	0.017	Office SBP, mmHg	1.057	(1.013–1.102)	0.010
ACEI/ARB (yes vs. no)	0.600	(0.319–1.128)	0.113	ACEI/ARB (yes vs. no)	0.491	(0.109–2.206)	0.354
β-blocker (yes vs. no)	1.574	(0.861–2.878)	0.140	β-blocker (yes vs. no)	2.071	(0.491–8.739)	0.322
CCB (yes vs. no)	1.271	(0.637–2.535)	0.496	CCB (yes vs. no)	0.412	(0.097–1.747)	0.229
Thiazide (yes vs. no)	1.100	(0.563–2.151)	0.780	Thiazide (yes vs. no)	1.926	(0.474–7.826)	0.360
Furosemide (yes vs. no)	0.850	(0.106–6.822)	0.878	Furosemide (yes vs. no)	19.968	(1.274–312.928)	0.033
HDLC, mg/dL	0.986	(0.961–1.013)	0.314	HDLC, mg/dL	0.961	(0.902–1.024)	0.217
eGFR, mL/min /1.73 m^2^	1.023	(1.006–1.041)	0.008	eGFR, mL/min /1.73 m^2^	1.026	(0.990–1.063)	0.161
UA ≥7.5 mg/dL (yes vs. no)	2.789	(1.399–5.560)	0.004	UA ≥7.5 mg/dL (yes vs. no)	6.653	(1.395–31.737)	0.017

*ACEI, angiotensin converting enzyme inhibitor; ARB, angiotensin receptor blocker; BMI, body mass index; CCB, calcium channel blocker; CI, confidence interval; eGFR, estimated glomerular filtration rate; HDLC, high density lipoprotein cholesterol; HR, hazard ratio; SBP, systolic blood pressure; UA, uric acid*.

**Table 8:** Uric acid 7.0 mg/dL and decline of estimated glomerular filtration rate (eGFR).

**eGFR >25% reduction**	**HR**	**(95% CI)**	***P*-value**	**eGFR >50% reduction**	**HR**	**(95% CI)**	***P*-value**
**Univariate analysis**				**Univariate analysis**			
UA ≥7.0 mg/dL (yes vs. no)	1.133	(0.634–2.023)	0.673	UA ≥7.0 mg/dL (yes vs. no)	2.126	(0.649–6.969)	0.213
**Multivariate analysis**				**Multivariate analysis**			
Age, years	1.033	(1.006–1.060)	0.018	Age, years	1.018	(0.956–1.083)	0.584
Sex (male vs. female)	0.732	(0.382–1.403)	0.347	Sex (male vs. female)	0.298	(0.071–1.252)	0.098
BMI, kg/m^2^	0.996	(0.913–1.087)	0.929	BMI, kg/m^2^	0.904	(0.738–1.108)	0.330
Office SBP, mmHg	1.021	(1.004–1.039)	0.015	Office SBP, mmHg	1.055	(1.013–1.098)	0.009
ACEI/ARB (yes vs. no)	0.648	(0.347–1.209)	0.173	ACEI/ARB (yes vs. no)	0.601	(0.141–2.572)	0.493
β-blocker (yes vs. no)	1.451	(0.797–2.640)	0.223	β-blocker (yes vs. no)	1.581	(0.403–6.206)	0.511
CCB (yes vs. no)	1.303	(0.657–2.584)	0.449	CCB (yes vs. no)	0.513	(0.130–2.021)	0.340
Thiazide (yes vs. no)	1.080	(0.540–2.160)	0.829	Thiazide (yes vs. no)	1.847	(0.442–7.720)	0.400
Furosemide (yes vs. no)	0.893	(0.111–7.156)	0.915	Furosemide (yes vs. no)	16.980	(1.243–232.037)	0.034
HDLC, mg/dL	0.985	(0.959–1.011)	0.259	HDLC, mg/dL	0.961	(0.905–1.021)	0.195
eGFR, mL/min /1.73 m^2^	1.019	(1.001–1.036)	0.034	eGFR, mL/min /1.73 m^2^	1.017	(0.981–1.054)	0.354
UA ≥7.0 mg/dL	1.577	(0.803–3.095)	0.186	UA ≥7.0 mg/dL	2.756	(0.607–12.519)	0.189

*ACEI, angiotensin converting enzyme inhibitor; ARB, angiotensin receptor blocker; BMI, body mass index; CCB, calcium channel blocker; CI, confidence interval; eGFR, estimated glomerular filtration rate; HDLC, high density lipoprotein cholesterol; HR, hazard ratio; SBP, systolic blood pressure; UA, uric acid*.

The subgroup analysis by gender was further conducted. A baseline UA level ≥8.0 mg/dL was associated with a risk of >25% decline in eGFR in both female (HR: 5.658; 95% CI: 1.244–25.747, *P* = 0.025) and male (HR: 2.798; 95% CI: 1.147–6.825, *P* = 0.024). As we further lower the cut-off value, a baseline UA level ≥7.5 mg/dL was associated with a risk of >25% decline in eGFR in male (HR: 2.374; 95% CI: 1.013–5.559, *P* = 0.047), but not in female (HR: 3.454; 95% CI: 0.895–13.332, *P* = 0.072). As for the major renal event, a baseline UA level ≥8.0 mg/dL was associated with a >50% decline in eGFR in female (HR: 40.086; 95% CI: 2.606–616.712, *P* = 0.008), but not in male (HR: 7.320; 95% CI: 0.476–112.592, *P* = 0.153). However, *P*-values for interaction were all insignificant (Table 9).

**Table 9:** Uric acid levels and decline of estimated glomerular filtration rate (eGFR) in female and male.

**eGFR25% decline**	**Female**			**Male**			
	**HR**	**(95% CI)**	***P*-value**	**HR**	**(95% CI)**	***P*-value**	***P* for interaction**
UA ≥8.0 mg/dL (yes vs. no)^*^	5.658	(1.244–25.747)	0.025	2.798	(1.147–6.825)	0.024	0.373
UA ≥7.5 mg/dL (yes vs. no)^*^	3.454	(0.895–13.332)	0.072	2.374	(1.013–5.559)	0.047	0.381
UA ≥7.0 mg/dL (yes vs. no)^*^	1.792	(0.472–6.800)	0.391	1.290	(0.549–3.032)	0.559	0.461
**eGFR50% decline**	**Female**			**Male**			
	**HR**	**(95% CI)**	***P*** **-value**	**HR**	**(95% CI)**	***P*** **-value**	***P*** **for interaction**
UA ≥8.0 mg/dL (yes vs. no)^*^	40.086	(2.606–616.712)	0.008	7.320	(0.476–112.592)	0.153	0.681
UA ≥7.5 mg/dL (yes vs. no)^*^	7.269	(0.758–69.757)	0.086	308.437	(0.233–407815.643)	0.118	0.614
UA ≥7.0 mg/dL (yes vs. no)^*^	4.427	(0.422–46.401)	0.215	24.980	(0.761–820.370)	0.071	0.809

*CI, confidence interval; HR, hazard ratio; UA, uric acid*.

* *Adjusted for age, BMI, office SBP, ACEI/ARB, β-blocker, CCB, thiazide, furosemide, HDLC, and eGFR*.

## Discussion

This study aimed to investigate the relationship between baseline serum UA levels and renal outcomes in patients with hypertension. Our investigation suggests that hyperuricemia, with a cutoff of 7.5 or 8.0 mg/dL, is related to the decline of renal function in Han Chinese hypertensive patients in Taiwan.

Several modifiable and unmodifiable mediators are related to the development and progression of CKD (18, 19). Among them, hypertension was one of the most important contributors to CKD (1, 2). In some hypertensive patients, however, renal function continued to deteriorate progressively even when the BP was under control. In our previous study, 11.2% of hypertensive patients still suffered from renal function decline when their BP was controlled to <140/90 mmHg (17). It is essential to identify specific characteristics that increase the risk of renal insufficiency in this population. Therefore, we focused on another possible modifiable risk factor, hyperuricemia.

Several studies have indicated that hyperuricemia is a predictor of the occurrence of renal disease in the general population. Two community cohorts in the United States, which involved 13,338 participants with 8.5 years of follow-up, suggested that elevated UA levels were an independent risk factor for incident kidney disease (3). Another mass community-based screening conducted in Japan, with 48,177 participants, further identified UA level as a major factor for ESRD in females during 7 years. The study showed that the incidence of ESRD per 1,000 women was 0.87 for those without hyperuricemia and 9.03 for those with hyperuricemia, with a hazard ratio of 5.77 (4).

In addition to the development of kidney diseases, high UA levels have been shown to exacerbate the progression of renal impairment, including diabetic nephropathy (5–7), IgA nephropathy (20–22), nephrosclerosis (23), and allograft nephropathy (24, 25). However, few studies have focused on patients with hypertension. The Uric Acid Right for Heart Health (URRAH) project, a cross-sectional study with 26,971 Italian patients with 62% being hypertensive patients, indicated that those with CKD were 10 times more likely to have hyperuricemia than those with intact renal function (8). Whether hyperuricemia presented as the cause, co-existing factor, or consequence of CKD was not investigated in this observational study. On the other hand, a 4.8-year cohort study in Japan demonstrated that UA level was not an independent risk factor for ESRD in hypertensive nephropathy (9). However, the follow-up period might be insufficient for progression to ESRD, making the results unremarkable. On the contrary, our prospective study emphasized early prevention of CKD in hypertensive patients by defining renal events as >25% and >50% reduction in eGFR.

Despite the strong association referred to by the above epidemiological data, the precise pathogenetic mechanism for urate nephropathy has not been well-established. It was hypothesized that the deposition of urate crystals in the medullary interstitium induced an inflammatory response, potentially leading to interstitial fibrosis and eventually CKD (26–28). The histological changes, including needle-like birefringent crystals of urate along with vascular sclerosis and tubular atrophy, provided evidence for urate nephropathy (29). However, both the pathological evidence and clinical manifestations were non-specific, making it difficult to differentiate it from other common etiologies, such as diabetic nephropathy. Whether hyperuricemia serves as a marker or contributor to renal injury is still under debate (30–32). To clarify the association between the UA level and renal outcome, we included patients with relatively preserved renal function at baseline (eGFR of 86.0 ± 19.4 mL/min/1.73 m^2^) and excluded those with diabetes mellitus. Other possible causes that affected renal function, including smoking (33), metabolic syndrome (34), and use of fibrate, statin or other medication (35, 36) were analyzed as well. The significant results of our study implied that hyperuricemia contributes to renal impairment.

There is no consensus on the target UA level in either the general population or patients with hypertension. The American College of Rheumatology Guideline suggested initiating intervention in patients with first gout flare only when the UA level exceeds 9.0 mg/dL, targeting a UA level <6.5 mg/dL (13). The European Alliance of Associations for Rheumatology proposed a stricter goal with initiating treatment in those with UA >8.5 mg/dL and targeting a UA level <6.0 mg/dL (14). However, the above recommendation applies only to patients with gout and does not address the impact of hyperuricemia on renal disease or hypertensive patients. Several studies have aimed to provide cutoff values for the prediction of renal disease. A study conducted in Vienna, with 21,475 healthy volunteers and a 7-year follow-up period, referred that the odds ratio for the development of renal insufficiency (eGFR <60 mL/min per 1.73 m^2^) increased dramatically when UA level exceeded 7.0 mg/dL in women and 8.0 mg/dL in men. The UA level between 7.0 and 8.9 mg/dL was associated with a nearly doubled risk for incident kidney disease and those with UA levels >9.0 mg/dL had a tripled risk (37). Another study that enrolled patients with nephrosclerosis suggested that the optimal UA cutoff value for predicting an eGFR decline by >50% from baseline or ESRD was 8.0 mg/dL (23). In our investigation, the reduction of eGFR was not observed in patients with UA >7.0 mg/dL but was significant if the cutoff value was set at 7.5 mg/dL or higher. This result served as important information for both physicians and patients in predicting the future risk of renal diseases. By initiating the evaluation earlier, we hope to delay the development of CKD in patients with hypertension.

The definition for hyperuricemia is gender-specific (10, 12). Therefore, whether there are different UA thresholds for predicting renal impairment in male and female is of our interest. One previous study suggested that the risk for incident kidney disease was associated with gender. The risk increased as UA level exceeded 6 to 7 mg/dL in women and 7 to 8 mg/dL in men (37). Our subgroup analysis seemed to provide gender-specific UA cutoff value as well, female as 8.0 mg/dL and male as 7.5 mg/dL, for minor renal event. However, none of the interaction tests was significant. This finding was consistent with one meta-analysis, which revealed no difference between men and women in UA level and CKD (38).

Despite numerous studies indicating the association between UA levels and renal diseases, data on the effects of uric acid-lowering agents on renal outcomes are limited and inconsistent. Three randomized, controlled trials, conducted in Hong Kong, Spain, and Iran, respectively, revealed that fewer patients in the allopurinol group endorsed renal function deterioration compared to the control group (39–41). However, several studies have shown different outcomes. The CKD-FIX Study (randomized Controlled trial of slowing of Kidney Disease progression From the Inhibition of Xanthine oxidase), enrolling a total of 363 patients with stage 3 or 4 CKD, concluded that allopurinol did not appear to effectively alter the progression of renal insufficiency during a 2-year follow-up (42). One of the possible explanations for this opposite result is that the study did not include UA level–based criteria at enrollment. Therefore, some participants had normal UA levels, while others had elevated UA levels. On the other hand, when comparing different urate-lowering agents, febuxostat reduced UA more and earlier than allopurinol (43). However, there was no difference in the decline of renal function between the two groups during a 3-year period (44). Therefore, additional comprehensive trials involving a larger cohort of participants to determine the long-term efficacy of different urate-lowering agents, as well as to characterize sub-populations who would benefit from urate-lowering agents, would be essential.

### Study Limitations

This study has several limitations that must be addressed. First, this was an observational study. There may have been a selection bias in patient enrollment. However, we tried our best to exclude participants with diabetes and other comorbidities to attenuate the impacts of other factors related to renal function deterioration. Second, the number of participants and major renal events was relatively small. Though only 11 participants experienced major renal events, there were 52 participants meet the criteria of minor renal events. The impacts of baseline UA levels on major and minor events were consistent, which increased the strengths of our study. Further studies with large sample size will be indicated. Third, our study was conducted only in Chinese patients with hypertension in Taiwan. Since UA levels may vary between different ethnic backgrounds (45, 46), our findings should be tested in hypertensive patients with different ethnic backgrounds in the future. Fourth, we did not investigate the impact of uric acid-lowering agents on renal function. To better clarify the relationship between UA levels and renal function, patients treated with urate-lowering agents were excluded from our study. However, whether hypertensive patients would benefit from early intervention for hyperuricemia is unknown. Therefore, further interventional trials should be conducted to determine the efficacy of urate-lowering agents for renal protection. Finally, although the hypertensive patients in our cohort were educated for dietary modification during the out-patient clinic follow-up, we did not have detail information about the dietary. Further studies with detail dietary information will be indicated.

## Conclusion

A high serum UA level is a significant risk factor for the decline in renal function in Han Chinese hypertensive patients in Taiwan. Patients with a baseline UA level ≥7.5 mg/dL were associated with minor or major nephropathy. Our findings support the routine measurement of serum UA levels in hypertensive patients to identify those who are more susceptible to the development of nephropathy. However, further studies are needed to clarify whether early intervention with urate-lowering agents could prevent renal impairment in hypertensive patients.

## Data Availability Statement

The raw data supporting the conclusions of this article will be made available by the authors, without undue reservation.

## Ethics Statement

The studies involving human participants were reviewed and approved by the Ethics Committee of Taipei Veterans General Hospital. The patients/participants provided their written informed consent to participate in this study.

## Author Contributions

Y-HH, C-CH, and L-YL: conceptualization. C-CH: methodology and formal analysis, resources and data curation, writing—review and editing, project administration, and funding acquisition. Y-HH, L-YL, and J-WC: writing—original draft preparation. All authors contributed to the article and approved the submitted version.

## Funding

This research was funded by research grant V110C-058, V111C-086, and V111EA-014 from Taipei Veterans General Hospital, Taipei, Taiwan, R.O.C., and research grant MOST 108-2314-B-075-062-MY3 from the Ministry of Science and Technology, Taiwan, R.O.C.

## Conflict of Interest

The authors declare that the research was conducted in the absence of any commercial or financial relationships that could be construed as a potential conflict of interest.

## Publisher's Note

All claims expressed in this article are solely those of the authors and do not necessarily represent those of their affiliated organizations, or those of the publisher, the editors and the reviewers. Any product that may be evaluated in this article, or claim that may be made by its manufacturer, is not guaranteed or endorsed by the publisher.
